# Malignant colo-duodenal fistula; case report and review of the literature

**DOI:** 10.1186/1477-7819-4-86

**Published:** 2006-12-05

**Authors:** Ruth Soulsby, Edmund Leung, Nigel Williams

**Affiliations:** 1 Department of General Surgery, University Hospitals Coventry, Clifford Bridge Road, Walsgrave, CV2 9DX, UK

## Abstract

**Background:**

Colo-duodenal fistula is a rare complication of malignant and inflammatory bowel disease. Cases with malignant colo-duodenal fistulae can present with symptoms from the primary, from the fistula or from metastatic disease. The fistula often results in diarrhoea and vomiting with dramatic weight loss. Upper abdominal pain is usually present as is general malaise both from the presence of the disease and from the metabolic sequelae it causes. The diarrhoea relates to colonic bacterial contamination of the upper intestines rather than to a pure mechanical effect. Vomiting may be faeculant or truly faecal and eructation foul smelling but in the case reports this 'classic' symptomatology was often absent despite a fistula being present and patent enough to allow barium through it. Occasionally patients will present with a gastro-intestinal bleed.

**Case presentation:**

We present an unusual case of colorectal carcinoma, where a 65 year old male patient presented with diarrhoea and vomiting secondary to a malignant colo-duodenal fistula near the hepatic flexure. Adenocarcinoma was confirmed on histology from a biopsy obtained during the patient's oesophageogastroduodenoscopy, and the fistula was demonstrated in his barium enema. Staging computed tomography showed a locally advanced carcinoma of the proximal transverse colon, with a fistula to the duodenum and regional lymphadenopathy. The patient was also found to have subcutaneous metastasis. Following discussions at the multidisciplinary meeting, this patient was referred for palliation, and died within 4 months after discharge from hospital.

**Conclusion:**

We present the case, discuss the management and review the literature. Colo-duodenal fistulae from colonic primaries are rare but early diagnosis may allow curative surgery. This case emphasises the importance of accurate staging and repeated clinical examination.

## Background

Patients with colorectal cancer usually present in the 6^th ^to 8^th ^decade with symptoms such as change of bowel habit, bleeding per anum, passage of mucus and abdominal discomfort. Anorexia and weight loss may occur if the tumour mass is large or if it becomes disseminated [1].

We present an unusual case of colorectal carcinoma, where the patient presented with diarrhoea and vomiting secondary to a malignant colo-duodenal fistula near the hepatic flexure. We present the case, discuss the management and review the literature.

## Case presentation

A 65 year-old male presented to the hospital with a two week history of diarrhoea and vomiting. He had no abdominal pain and no symptoms or signs of gastro-intestinal blood loss. He was initially thought to have gastroenteritis, stool samples were sent and he was treated conservatively. He was noted to be mildly anaemic with a haemoglobin level of 10.7. A flexible sigmoidoscopy (FS) was performed to begin investigation of his diarrhoea. This showed a small polyp which was benign on histology and no other abnormality. He settled and was then discharged home. Barium enema (BE) was booked as an outpatient investigation to assess the rest of the colon, and if negative, oesophageogastroduodenoscopy (OGD) would then be performed to investigate his anaemia. The waiting list for colonoscopy was too lengthy at that time, and the combination of BE and FS routinely replaced colonoscopy.

The patient was readmitted three weeks later in hypovolaemic shock with a blood pressure of 88/56, pulse 110. During this admission, he was noted to be cachetic, weighing just over 55 kilograms (Body mass index = 16.2). On further questioning he had lost over 12 kilograms in weight since the onset of his vomiting several weeks before. His admission haemoglobin level on this admission was 7.5.

**Figure 1:**
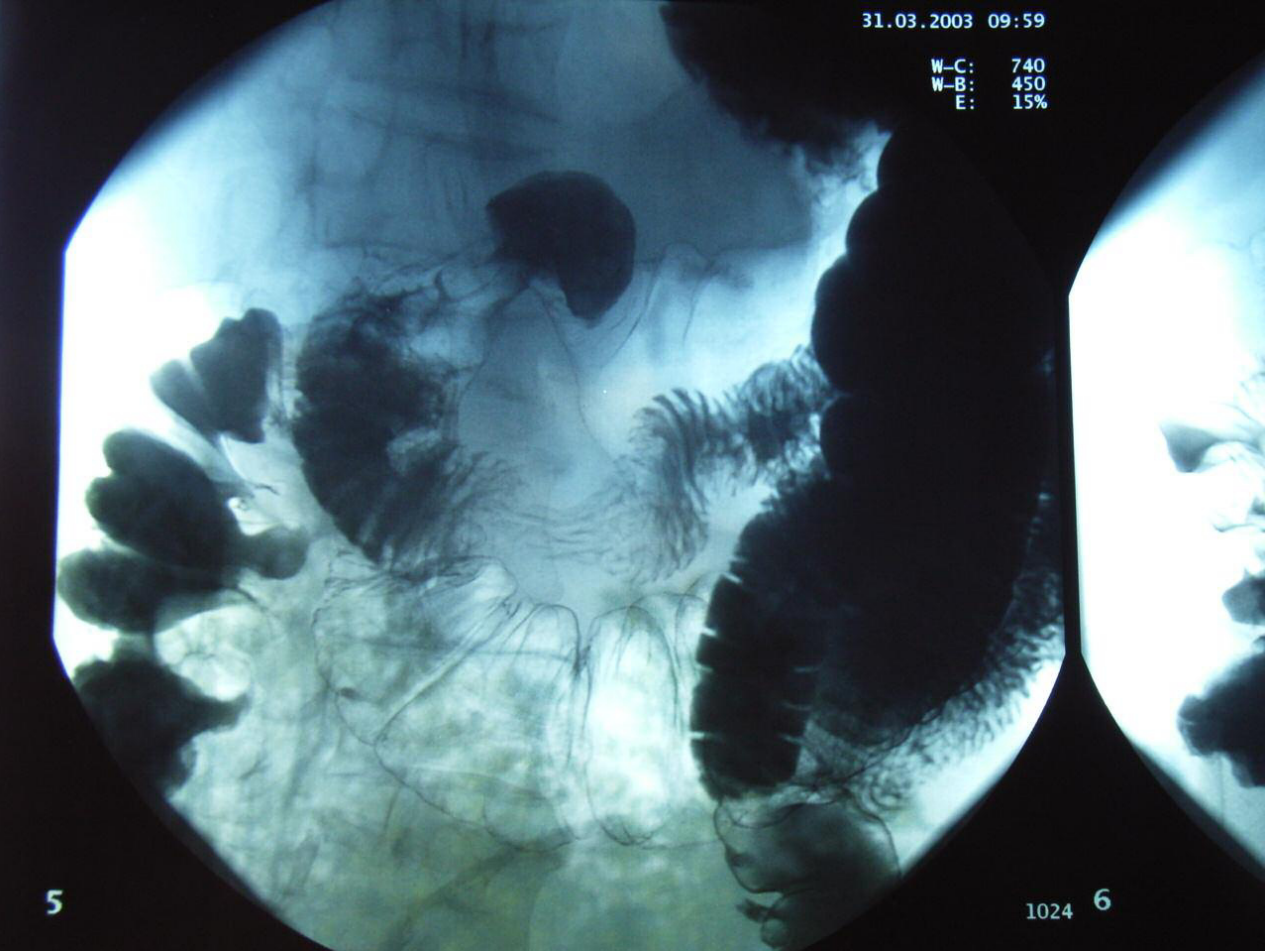
Barium enema study showing leakage of barium from the hepatic flexure into duodenum.

In view of the worsening anaemia on this admission and a past history of duodenal ulcer in 1996, in-patient OGD and BE were requested. Meanwhile, the patient continued to receive intravenous fluid, blood and acid suppressing agents. OGD revealed an opening in the second part of the duodenum, which was biopsied. Histology showed poorly differentiated adenocarcinoma and one piece of normal colonic mucosa. BE clearly demonstrated a fistula between the hepatic flexure and second part of duodenum (figure 1), and the patient vomited the barium following his enema.

**Figure 2:**
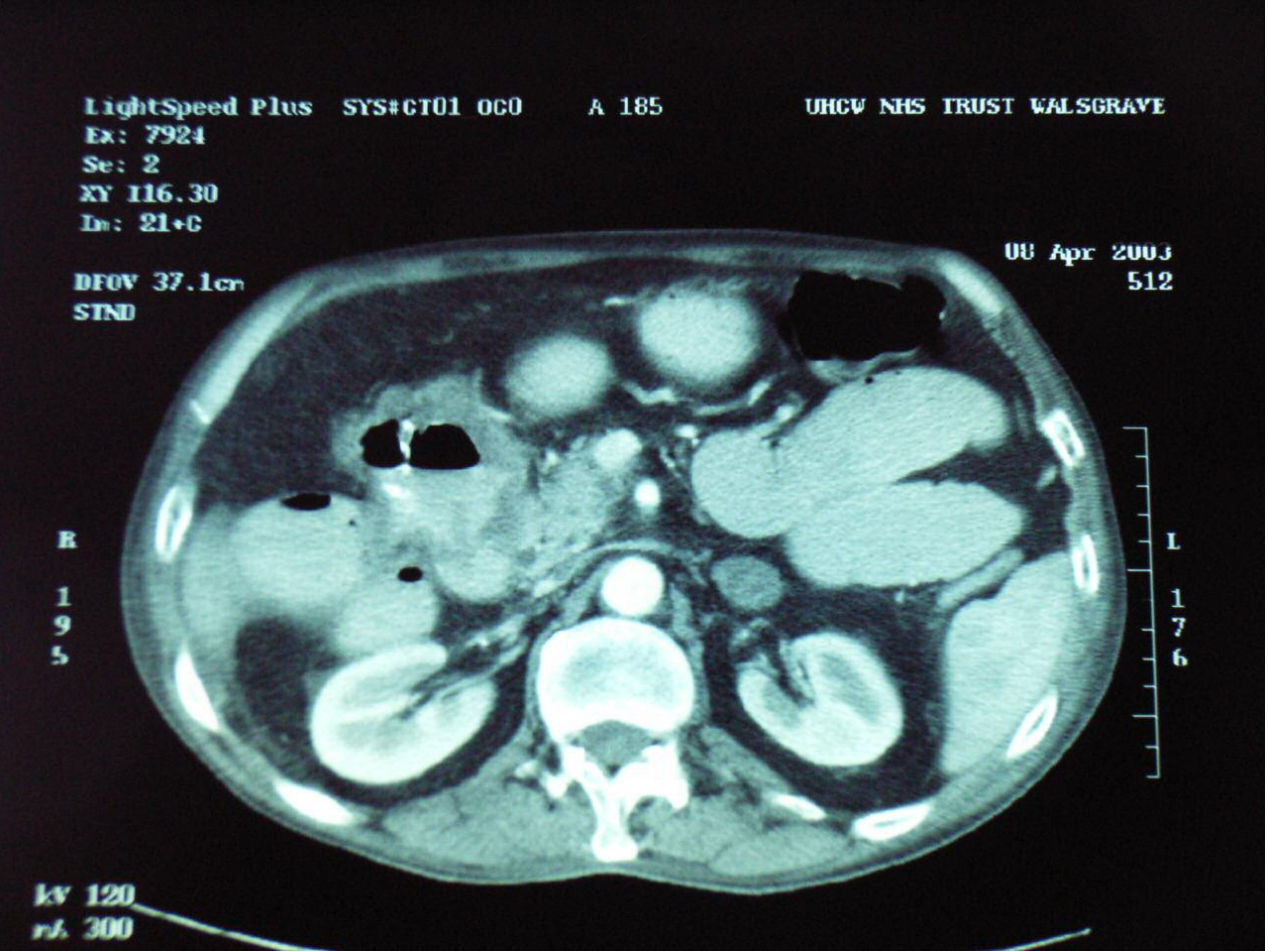
Abdominal CT showing a locally advanced tumour in proximal transverse colon with a fistula into the second part of duodenum.

Subsequent isotope bone scan and liver ultrasound did not show any signs of metastasis. Computed tomography (CT) of the abdomen showed a locally advanced carcinoma of the proximal transverse colon (figure 2), with a fistula to the duodenum. The regional lymph nodes were noted to be enlarged and there was enlargement of the left adrenal of uncertain significance. There was also a small volume of ascites.

**Figure 3:**
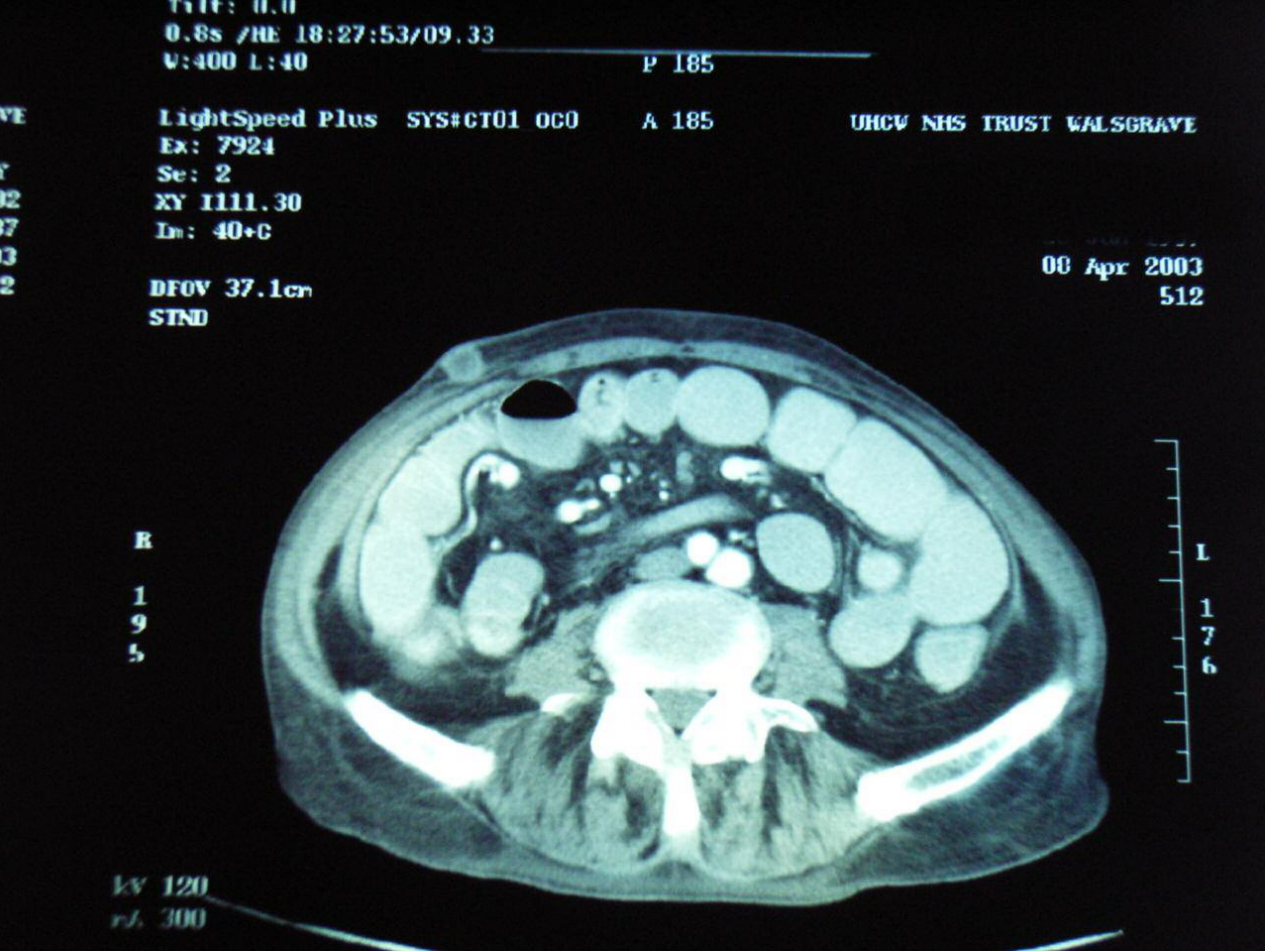
Abdominal CT section showing a subcutaneous nodule on the right abdominal wall.

Clinically during the patients admission a subcutaneous nodule developed on the right abdominal wall. The mass was erythematous, non-tender, mobile and deep to skin. Fine needle aspiration cytology confirmed subcutaneous metastasis of poorly differentiated adenocarcinoma (figure 3). Over the next few weeks a similar subcutaneous metastasis developed over his left scapular area.

The patient's nutritional status was corrected. His vomiting had stopped. It was explained to him and his family that surgery was inappropriate. He was discussed at the multidisciplinary meeting but the feeling was that chemotherapy would serve only to enlarge the fistula and be likely to exacerbate his symptoms without any great benefit to him. Radiotherapy directed at the subcutaneous lesions was utilised as the only feasible option of palliation. A palliative ileostomy with a feeding jejunostomy was considered, however, the patient was reluctant with the prospect of having an ileostomy especially his symptoms were well controlled. His care was subsequently transferred to a palliative care unit. The patient died 4 months after discharge from hospital.

## Discussion

It is unusual, however, for colon cancers to invade the duodenum to such an extent that a malignant fistula is created. The first case report of a colo-duodenal fistula was by Haldane in 1862 reporting in the Edinburgh Medical Journal [2]. His patient had a malignant fistula arising from the hepatic flexure. A report of 1,400 cases of right colon cancer reported nearly a century later showed that in this number there were only two malignant colo-duodenal fistulae noted [3]. There were six cases of duodenal invasions without evidence of fistulation. Reports of malignant fistulae are sporadic in the literature. They are usually secondary to a colonic primary rather than an upper gastro-intestinal malignancy and due to their rarity are usually reported as a single case.

The biggest series was reported in 1951 in which a large series of colo-duodenal fistulae were reported. Hershenson documented only one case among 8,100 autopsies [4]. One report estimated the incidence of duodenocolic fistula in the United States to be 1 in 900 colorectal carcinomas [5]. Most other reports have been 4 patients or fewer [6-8]. One article reported two cases, both of which had synchronous cancers that had to be removed en-bloc [9]. It would be important to look for these if curative surgery was planned.

Colo-duodenal fistulas are associated with advanced carcinomas of the hepatic flexure. In present days, these fistulae rarely occur because of an earlier discovery and resection of the tumour. Therefore, only isolated reports appeared. These malignant causes, other than colonic carcinoma, include carcinoma of the gall bladder [10,11] carcinoma of the duodenum [12] and even from metastatic disease of the oesophagus [13].

Benign causes reported include benign duodenal ulceration [14], Crohn's disease [15-19], gallstones [20-22], a pancreatic pseudocyst rupture [23-25] and stent migration [26]. Rarer causes have been noted due to tuberculous disease, typhoid ulceration and non specific inflammatory lesions. They have also been reported secondary to complicated appendicitis. Some patients have developed fistulae as a post-operative event. A ruptured duodenal diverticulum was the first report of a benign cause for colo-duodenal fistula in 1863 [27]. Spontaneous colo-duodenal fistulae have also been reported with no obvious aetiology. A case series of three patients presented with chronic diarrhoea and malabsorption. They all showed subtle tracts which would have been missed if their presence had not been found radiologically previously. All three patients had excision of the tract and settled post-operatively. These fistulae were always from the mid transverse to the duodenum and not related to inflammatory bowel disease – the pathology was non-specific and the authors postulated a congenital cause [28].

Patients with malignant duodenal fistulae can present with symptoms from the primary, from the fistula or from metastatic disease. The fistula often results in diarrhoea and vomiting with dramatic weight loss. Upper abdominal pain is usually present as is general malaise both from the presence of the disease and from the metabolic sequelae it causes. The diarrhoea relates to colonic bacterial contamination of the upper intestines rather than to a pure mechanical effect [29]. It has also been suggested that duodenal bile salts have an irritating effects on colonic mucosa resulting in diarrhoea [30]. Vomiting may be faeculant or truly faecal and eructation foul smelling but in the case reports this 'classic' symptomatology was often absent despite a fistula being present and patent enough to allow barium through it. Occasionally patients will present with a gastro-intestinal bleed.

Radiology is useful to delineate the fistula as the difference in surgical management between a gastro-colic and a duodeno-colic fistula is profound. In the reports, barium enema seemed more likely to delineate the fistula than barium meals. More recently, CT scanning is of great value in assessing metastatic spread as well as assessing the local invasion of the primary.

Treatment of malignant colo-duodenal fistulae depends on the extent of the primary tumour, the presence of metastatic disease and the general condition of the patient. It is often necessary to spend time rehydrating and transfusing the patient and correcting the, often profound, electrolyte disturbances. Some patients present with gross weight loss from malnutrition (not just dehydration). Malnutrition is due to the malabsorptive state that the bacterial overgrowth causes in the small bowel [29]. These patients may benefit from pre-operative total parenteral nutrition (TPN) [6]. Other authors advocate surgery as soon as feasible [30].

The complexity of the pancreatoduodenal area makes the operative approach challenging [30]. There are various curative operations reported, all of which include a right hemicolectomy. Chang treated 20 of his patients with right hemicolectomy with partial duodenectomy and *primary closure of the duodenal wall defect *[33]. His mortality was 28%, mainly attributed to leakage from the duodenal defect and local recurrence. Ellis described using a *jejunal loop to close the duodenal wall defect *[31]. In his series of 6 patients of whom 2 had a fistula and 4 had direct invasion without fistula formation. The survival with non-fistulating patients was better. In 1944, Linton first described the two-stage procedure consisting of defunctionalization of the fistula by gastrojejunostomy and ileotransverse colostomy as the first stage, followed by *tumour resection and pancreaticoduodenectomy*, allowing nutritional replacement between the two stages [34]. Nowadays, the one-stage procedure is more commonly adopted, because of advances in perioperative intensive care and availability of TPN [32]. Results between one-stage and two-stage procedure were similar and authors are divided on their approach as to whether to operate in a one-stage manner or to try a two-stage procedure [6,8,29,31].

There are reports of patients having a curative resection with en bloc pancreaticoduodenectomy with reasonable survival – although the numbers are small [35]. Many other reports also support radical resection whenever feasible as treatment of choice [5,9]. Izumi reviewed a series of 34 cases of malignant colo-duodenal fistulae in Japan, and their survival with with en bloc pancreaticoduodenectomy ranged from 7 days to 4 years (median = 10 months, 19F:15M) [36]. However, Hirsch reported on a patient following the two-stage procedure and survived for 26 years [37].

The prognosis depends on staging of the disease at the time of diagnosis. If the malignancy is too extensive, curative resection may not be possible. Ileotransverse colostomy with gastrojejunostomy has been described [38]. The intestinal shunting would give relief of symptoms caused by the fistula, but could not prevent bleeding or other paraneoplastic symptoms caused by the tumour itself. Some authors advise utilizing the exclusion bypass principle in specially selected patients [29]. This principle entailed isolating the affected portions of the colon from the remaining large intestine and anastomosing it to itself. Therefore, bacterial contamination of the upper tract would be reduced and potential closed-loop obstruction could be avoided. The survival of patients with malignant colo-duodenal fistula is usually less than 12 months when treated with such palliative operations as ileotransverse colostomy with gastrojejunostomy [3,5].

Our patient presented with symptoms from his primary and his fistula, he had gross weight loss, diarrhoea and vomiting. He required vigorous rehydration and transfusion. OGD both confirmed the duodenal fistula and allowed biopsies to be taken. It was thought initially that he may have had operable local disease. Lymphadenopathy *per se *on CT scanning, when related to a colonic primary, does not necessarily mean they are involved. Some reports suggest the sensitivity for detection of malignant lymphadenopathy is only about 45% in colonic cancer [35]. We would therefore have needed histology from his adrenal (as incidental lesions are relatively common).

However, during his short stay repeated examination revealed his rapidly expanding subcutaneous metastasis. This was relatively easily assessed with fine needle aspiration cytology and when this proved positive, with poorly differentiated adenocarcinoma, it was clear that surgery was inappropriate, even for palliative purposes. It was then possible to focus his care in a palliative direction.

## Conclusion

Colo-duodenal fistulae from colonic primaries are rare but it is important to identify these preoperatively as en bloc resection with curative intent may well require a pancreatcoduodenectomy. This case emphasises the importance of accurate staging and repeated clinical examination.

## Conflict of interest

The author(s) declare that they have no competing interests.

## Authors' contributions

**RS **wrote the case report and performed literature search

**EL **reviewed literature and revised the manuscript

**NW **was the surgeon managing the patient and reviewed the manuscript

All authors have read and approved the manuscript.
